# WIP1 phosphatase as pharmacological target in cancer therapy

**DOI:** 10.1007/s00109-017-1536-2

**Published:** 2017-04-24

**Authors:** Soňa Pecháčková, Kamila Burdová, Libor Macurek

**Affiliations:** Department of Cancer Cell Biology, Institute of Molecular Genetics of the ASCR, CZ-14220 Prague, Czech Republic

**Keywords:** Cancer, Phosphatase, Checkpoint, DNA damage response, Inhibitor, p53

## Abstract

DNA damage response (DDR) pathway protects cells from genome instability and prevents cancer development. Tumor suppressor p53 is a key molecule that interconnects DDR, cell cycle checkpoints, and cell fate decisions in the presence of genotoxic stress. Inactivating mutations in *TP53* and other genes implicated in DDR potentiate cancer development and also influence the sensitivity of cancer cells to treatment. Protein phosphatase 2C delta (referred to as WIP1) is a negative regulator of DDR and has been proposed as potential pharmaceutical target. Until recently, exploitation of WIP1 inhibition for suppression of cancer cell growth was compromised by the lack of selective small-molecule inhibitors effective at cellular and organismal levels. Here, we review recent advances in development of WIP1 inhibitors and discuss their potential use in cancer treatment.

## Introduction

Genetic information is continuously endangered by erroneous DNA metabolism as well as by various environmental factors that include ionizing radiation or chemotherapy representing two major non-surgical approaches in cancer therapy. Cells respond to genotoxic stress by activation of a conserved DNA damage response pathway (DDR) that abrogates cell cycle progression and facilitates DNA repair. This safeguard mechanism represents an intrinsic barrier preventing genome instability and protecting cells against tumor development [1–4]. Depending on the mode and level of DNA damage, DDR signaling network promotes temporary cell cycle arrest (checkpoint), permanent growth arrest (senescence), or programmed cell death (apoptosis). Genes coding for proteins involved in DDR are typically tumor suppressors and are commonly mutated in cancer. The DDR pathway is regulated by a spatiotemporally controlled cascade of posttranslational modifications of key proteins including protein phosphorylation and ubiquitination [5]. Following DNA damage, upstream protein kinases ATM and ATR are activated and spread the signal through phosphorylation of downstream transducing kinases CHK2 and CHK1 to rapidly establish the checkpoint arrest. Subsequently, checkpoint is reinforced by activation of the tumor suppressor protein p53 and its transcriptional target p21 that inactivates cyclin-dependent kinases.

After completion of DNA repair, activity of the DDR pathway is terminated by protein phosphatases that allow checkpoint recovery and restart cell proliferation. Serine/threonine phosphatases of PP2C family are evolutionary conserved negative regulators of cell stress response pathways and function as monomeric enzymes comprising of a conserved N-terminal phosphatase domain and non-catalytic C-terminal part [6]. Protein phosphatase 2C isoform delta is ubiquitously expressed at basal levels and its expression is strongly induced after exposure of cells to genotoxic stress in a p53-dependent manner (hence its alternative name WIP1 for wild-type p53-induced protein 1) [7]. Substrate specificity of the chromatin-bound WIP1 matches the phosphorylation sites imposed by ATM kinase, and thus, WIP1 can efficiently dephosphorylate p53, γH2AX, and possibly also other proteins involved in DDR [8, 9]. Downregulation of WIP1 by RNA interference leads to prolongation of the G2 checkpoint whereas overexpression of WIP1 causes checkpoint override [10, 11]. WIP1 phosphatase is overexpressed in multiple human cancers and was reported to act as oncogene. Conversely, loss of WIP1 delayed the onset of tumor development in mouse models [12–14]. Similarly, RNAi-mediated depletion of WIP1 inhibited cancer cell growth implicating WIP1 as promising pharmacological target [14]. Here, we discuss recent advances in development of a selective WIP1 inhibitor with proven efficiency in animal models and its potential use in cancer therapy.

## DNA damage response and role of WIP1 in checkpoint recovery

**Fig. 1:**
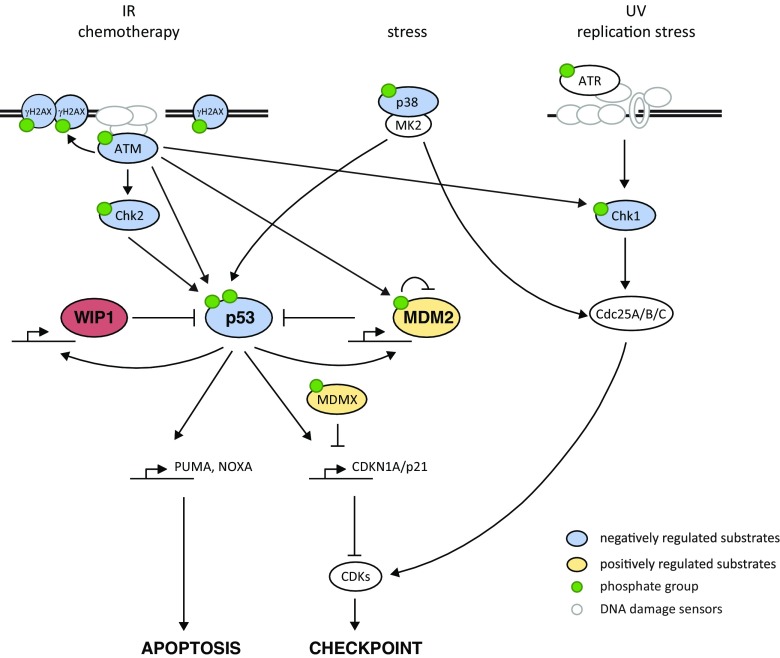
Role of WIP1 phosphatase in termination of DNA damage response. Exposed ssDNA caused by stalled replication forks or resected DSBs activates ATR/CHK1 pathway that targets CDC25 family of phosphatases, prevents activation of CDKs, and triggers cell cycle arrest. DSBs induced by ionizing radiation or chemotherapy activate ATM that orchestrates DNA repair by phosphorylating histone H2AX at chromatin and activates the cell cycle checkpoint. This is achieved by phosphorylation of p53 and Mdm2 that allows stabilization of p53 and triggers expression of CDKN1/p21. In addition, p53 stimulates expression of its negative regulators Mdm2 and WIP1. After accumulating sufficient protein levels, WIP1 inactivates p53 pathway and dephosphorylates other targets jointly contributing to termination of the DDR (negatively and positively regulated WIP1 substrates shown in *blue* and *yellow*, respectively). Persistent genotoxic stress can continuously activate p53 leading to senescence. Very high activation of p53 pathway leads to expression of *PUMA* and *NOXA* and leads to cell death

Various kinds of genotoxic stress activate kinases of PI3-kinase like family, including activation of ATM by DNA double-strand breaks (DSBs) and ATR by exposed single-stranded DNA (ssDNA) at stalled replication forks or resected DSBs (Fig. 1). ATM and ATR phosphorylate the effector checkpoint kinases CHK2 and CHK1 that target phosphatases Cdc25A/B/C leading to inactivation of cyclin-dependent kinases (CDKs) and cell cycle arrest. Under basal conditions, p53 is degraded by the E3 ubiquitin ligase MDM2 and transcriptionally inactivated at promoters by its enzymatically inactive homolog MDMX [15–18]. Following DNA damage, p53 is posttranslationally modified by ATM/CHK2, ATR/CHK1, and various acetyltransferases leading to its stabilization and oligomerization, binding to promoters and triggering transcription of various target genes involved in cell cycle arrest, DNA repair, apoptosis, senescence, and metabolism [19, 20]. CDKN1/p21 is a transcriptional target of p53 and potent inhibitor of CDKs that promotes maintenance of the G1 and G2 checkpoint. In non-stressed cells, expression of CDKN1/p21 is repressed by transcription intermediary factor 1-beta (also called KAP1) [21]. Phosphorylation of KAP1 at Ser824 by ATM and at Ser473 by CHK1/2 induced by genotoxic stress allows de-repression of CDKN1/p21 and contributes to checkpoint activation [21, 22].

Besides arresting the cell cycle progression, ATM promotes DNA repair by phosphorylating histone variant H2AX at S139 (called γH2AX) in the flanking chromatin and plethora of other DNA repair proteins. γH2AX acts as a docking platform for various mediator proteins and ubiquitin ligases that jointly regulate recruitment of either 53BP1 or BRCA1 proteins to the close proximity of the DNA lesion and thus control the DNA repair pathway choice [23]. Whereas 53BP1 in complex with RIF1 blocks DSB resection and promotes non-homologous end joining, recruitment of BRCA1 stimulates resection and therefore facilitates homologous recombination (HR). After completion of DNA repair, cells recover from the checkpoint arrest and reenter the cell cycle. By targeting claspin, an important cofactor of ATR, PLK1 kinase terminates the activation of CHK1 and is essential for recovery from the G2 checkpoint [24]. In addition, various protein phosphatases directly reverse multiple phosphorylations imposed by ATM/ATR and CHK1/2 and thus contribute to timely inactivation of DDR [25]. In particular, protein phosphatase PP4 targets Ser473 of KAP1 and has been implicated in recovery from the G1 checkpoint [26]. In contrast, WIP1 is needed for recovery from the G2 checkpoint [11, 26]. Whereas expression of WIP1 is potentiated by p53, it acts as a strong negative regulator of p53 pathway thus forming a negative feedback loop that allows termination of p53 response after completion of DNA repair [11]. WIP1 inhibits p53 directly by dephosphorylating Ser15 and indirectly through the stimulation of its negative regulators MDM2 and MDMX [10, 27–30]. In fact, WIP1 activity is needed throughout the G2 checkpoint to limit the level of p53/p21 pathway activation and to prevent degradation of cyclin B and a permanent cell cycle exit [31, 32]. Similarly, WIP1 was shown to suppress DNA damage-induced apoptosis in different cell types [33–35]. Besides targeting p53 pathway, WIP1 contributes to termination of DDR by dephosphorylation of ATM at Ser1981 and γH2AX at chromatin [9, 36–38]. Other reported substrates of WIP1 include active forms of CHK1, CHK2, and p38 that reside mostly in nucleoplasm [10, 39, 40]. Although WIP1 can dephosphorylate these proteins in vitro or when overexpressed, the physiological role of the chromatin-bound WIP1 in targeting these pathways remains unclear. Similarly, WIP1 was reported to counteract phosphorylation of the p65 subunit of NF-κB at Ser536 but more data are needed to clarify to what extent WIP1 regulates NF-κB pathway in inflammation [41].

Function of WIP1 is controlled in context of the cell cycle. Expression of WIP1 protein is low in G1, peaks in S/G2, and decreases during mitosis [42]. WIP1 is phosphorylated at multiple residues within the catalytic domain during mitosis which promotes its degradation by APC/cdc20 in prometaphase [42]. Absence of WIP1 in mitosis may allow cells to recognize low levels of endogenous DNA damage present in condensed chromosomes. These sites are labeled by γH2AX during mitosis and they are repaired after mitotic exit in subsequent G1 phase. During interphase, WIP1 is constitutively phosphorylated at Ser54 and Ser85 by HIPK2 kinase that results in a rapid turnover of WIP1 [43]. Keeping basal levels of WIP1 low probably allows cells to fully activate DDR in the presence of genotoxic stress, whereas p53-dependent induction of WIP1 expression allows termination of DDR after completion of DNA repair.

## WIP1 phosphatase as an oncogene

About a half of human solid tumors exhibit somatic mutations in the *TP53* gene that cause a deficient response to genotoxic stress and are commonly associated with poor prognosis [44, 45]. On the other hand, tumors carrying wild-type *TP53* frequently accumulate mutations in other genes that functionally compromise the p53 pathway and thus potentiate cell proliferation. As described above, WIP1 phosphatase is a negative regulator of DDR pathway and enhanced activity of WIP1 can contribute to tumor development.

**Fig. 2:**
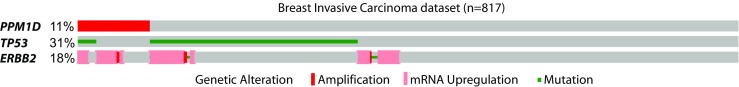
Amplification of *PPM1D* locus in breast cancer. Breast invasive carcinoma dataset (*n* = 817, [48]) was analyzed for *PPM1D* amplification (11%), *TP53* mutation (31%), and *ERBB2* amplification, overexpression, or mutation (18%) using cBioPortal [49]. Amplification of genes was analyzed using putative copy number alterations from GISTIC. Expression analysis was based on mRNA Expression z scores (RNA Seq V2 RSEM) where threshold was set at fourfold difference. Tendency to mutual exclusivity between *PPM1D* and *TP53* mutation as well as tendency to co-occurrence between *PPM1D* and *ERBB2* activation were statistically significant

WIP1 is encoded by *PPM1D* gene located at chromosomal locus 17q23.2 and its amplification was reported in about 10% of breast cancers [46, 47]. Importantly, amplification of *PPM1D* occurred significantly more often in breast tumors that retained wild-type *TP53* [46, 47] (Fig. 2). Similarly, common amplification of *PPM1D* was found in ovarian clear cell carcinoma, where mutations in *TP53* are relatively rare, but not in a more common serous carcinoma that typically contains mutated *TP53* [50, 51]. Besides breast and ovarian cancer, *PPM1D* copy numbers gain or overexpression at mRNA level were reported also in glioma, neuroblastoma, and medulloblastoma [47, 51–58]. High expression of WIP1 was also observed by immunohistological methods in a fraction of lung adenocarcinomas and gastric and colorectal cancers [55, 59, 60]. However, caution should be taken when interpreting the histopathological data, since none of the currently available antibodies was sufficiently validated in histological assays and the staining pattern does not correspond with expected nuclear localization of WIP1. Besides amplification, nonsense mutations occur in a hotspot region of the exon 6 of *PPM1D* [61, 62]. These point mutations of *PPM1D* result in expression of C-terminally truncated variants of WIP1 that exhibit higher protein stability and disable full activation of the checkpoint after genotoxic stress [62]. Besides breast and ovarian cancer, this type of mutations has been found in brainstem gliomas, lung adenocarcinoma, and prostate cancer [61–67]. WIP1 truncating mutations are considerably less common than *PPM1D* amplifications (usually below 1%) and their occurrence was reported to further increase after chemotherapy [66]. Although gain-of-function mutations in *PPM1D* efficiently suppress p53 function, their pathogenic role in cancer development still needs to be experimentally tested.

Amplification of *PPM1D* was initially suggested to promote breast cancer development through inactivation of the p53 and p38 MAPK pathways [52, 68]. In the same time, however, MMTV-driven overexpression of *PPM1D* in mice did not promote mammary tumor formation within 2 years suggesting that oncogenic properties of WIP1 may be relatively low [52, 69]. About one third of breast tumors with *PPM1D* overexpression showed also amplification of *ERBB2* suggesting that these two oncogenes may cooperatively promote breast cancer development [70]. Ablation of *PPM1D* in mice impaired spermatogenesis and decreased levels of B and T lymphocytes, both probably reflecting the decreased ability to respond adequately to endogenous DNA breaks occurring during meiosis or immunoglobulin gene rearrangements, respectively [71, 72]. Importantly, deletion of *PPM1D* strongly suppressed breast tumorigenesis in mice bearing MMTV-driven oncogenes *ERBB2* or *HRAS1* through the inactivation of p38 MAPK and p53 pathways [73]. Loss of *PPM1D* also dramatically delayed development of Eμ-myc-induced lymphomas in a p53-dependent manner [74]. In context of the colon, WIP1 was found to be highly expressed in the stem cell compartment and loss of *PPM1D* suppressed APC(Min)-driven polyp formation in mice suggesting that WIP1 might be involved also in development of colorectal cancer [75].

**Fig. 3:**
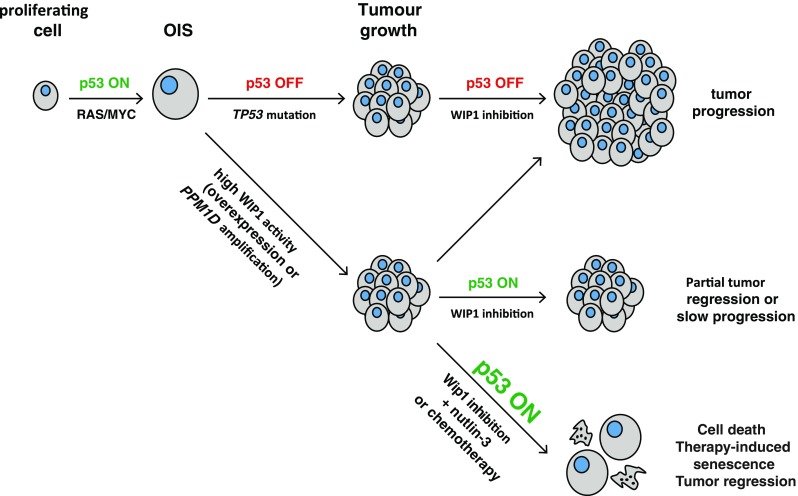
Model for WIP1 involvement in tumorigenesis and in therapeutic response. Activation of oncogenes (such as *RAS* and *MYC*) causes replication stress, stimulates p53 activity, and results in permanent cell cycle arrest called oncogene-induced senescence (OIS). Inactivating mutation of *TP53*, overexpression of WIP1, or amplification of *PPM1D* leads to suppression of p53 pathway, disables establishment of OIS, and promotes tumor formation. Inhibition of WIP1 does not affect proliferation of cancer cells with mutant *TP53* whereas it allows partial reactivation of p53 pathway in cells with wild-type *TP53* slowing down their proliferation. Combination of WIP1 inhibition with MDM2 antagonist nutlin-3 or with DNA damage-inducing chemotherapy allows maximal activation of p53 pathway leading to induction of cell death or senescence and preventing tumor growth

Exact molecular mechanism(s) by which WIP1 contributes to cell transformation still needs to be fully addressed. Data from the *PPM1D* knock-out mice and clinical specimens suggest a strong correlation between oncogenic behavior of WIP1 and the functional p53 pathway. In addition, gain-of-function mutations in *PPM1D* promote cell proliferation by overcoming p53 function, and conversely, loss of *PPM1D* slows down proliferation only in p53-proficient cells further supporting the model in which active WIP1 allows cells to overcome the tumor-suppressing barrier imposed by p53 pathway (Fig. 3). Whereas overexpressed WIP1 may not be sufficient to fully transform the cells, it can become more important under conditions of activation of oncogenes. It is well established that oncogene activation causes replication stress and induces senescence. An attractive possibility is that WIP1 may prevent oncogene-induced senescence and thus allow accumulation of mutations caused by proliferation under condition of replication stress. In addition, WIP1 was reported to regulate epigenetic changes in heterochromatin which may increase the C-to-T substitutions and thus contribute to genome instability [76]. Finally, overexpressed WIP1 was shown to impair DNA repair through nucleotide excision and base excision pathways [77, 78]. It should be noted that all these mechanisms by which WIP1 activity promotes genome instability are not mutually exclusive, and they may jointly contribute to tumorigenesis.

## Predicted structure of WIP1 phosphatase

Development of highly potent and specific small-molecule inhibitors is greatly facilitated by 3D structural data of the target proteins [79]. Since WIP1 structure has still not been determined, molecular models based on its homology with PPM1A (sharing ∼35% sequence identity) represent the only resource of information about WIP1 structure [80, 81]. Like the other PP2Cs, WIP1 acts as monomer consisting of the N-terminal catalytic domain (amino acids 1-375) and a presumably unstructured C-terminal tail [82]. Conserved negatively charged amino acids in the catalytic domain bind two Mg^2+^/Mn^2+^ ions and stabilize interaction of WIP1 with the phosphorylated substrate. A unique flap sub-domain resides in the catalytic domain close to the active site and can influence binding of different substrates by allosteric modulation [80]. Part of the flap domain is a basic amino acid-rich region (called B-loop; amino acids 235–268) that was proposed to bind to negatively charged phosphate on substrates [81]. In vitro studies established that WIP1 can specifically recognize two distinct substrate motifs, namely pSQ/pTQ (present in ATM, p53, MDM2, γH2AX, Chk1, Chk2) and pTxpY (present in the active form of p38 MAPK) [8]. In comparison to other PP2Cs, catalytic domain of WIP1 contains a proline-rich region (Pro-loop) that was proposed to mediate protein-protein interactions. However, the Pro-loop is not evolutionary conserved and its function in control of WIP1 activity still remains unclear. Translocation of WIP1 to the nucleus is controlled by two nuclear localization sequences (NLS). One NLS resides in the C-terminus (amino acids 535–552), while the other is located within the catalytic domain (amino acids 247–250) [62, 83]. Presence of the two NLS sequences explains why the C-terminally truncated mutants of WIP1 localize normally in the nucleus.

## Small-molecule inhibitors of WIP1

Based on data from *PPM1D* knockout mice and also from RNAi-mediated depletion of WIP1 in cancer cell lines, WIP1 was proposed as potential pharmacological target [73–75]. Since the structure of WIP1 is still unknown, the potential inhibitors of WIP1 were found by high-throughput screening of extensive chemical libraries. During the last decade, several compounds antagonizing WIP1 activity were developed; however, only one of these inhibitors exhibits high specificity to WIP1 and shows promising results in preclinical analysis.

Inorganic compound arsenic trioxide (ATO) was shown to inhibit WIP1 in vitro (IC_50_ >100 μM) and WIP1 depletion promoted ATO-induced apoptosis, probably by enhancing activation of Chk2/p53 and p38 pathways [84]. However, other studies demonstrated that ATO induced apoptosis by targeting multiple signal transduction pathways, suggesting that its selectivity to WIP1 is low [85]. Compound M321237 was identified by screening of a chemical library based on its ability to inhibit WIP1 activity in vitro [86]. Cell viability assay showed that M321237 sensitized MCF7 cells to doxorubicin. In vivo experiments reveled that administration of M321237 decreased tumor volumes in xenograft models; however, the selectivity of M321237 towards WIP1 has never been validated. Similar screening approach led to identification of CCT007093 that inhibited WIP1 in vitro with IC_50_ = 8.4 μM [87]. Cell viability in the presence of CCT007093 was suppressed in p53-proficient cancer cells carrying amplified *PPM1D* [87]. On the other hand, CCT007093 suppressed UV-induced apoptosis in skin keratinocytes by preventing activation of JNK, suggesting low specificity of the inhibitor towards WIP1 [88]. In addition, CCT007093 was shown to suppress cell proliferation regardless of the presence of WIP1 in U2OS cells confirming an off-target effect of the inhibitor [89]. Further, treatment of cells with CCT007093 did not affect levels of p53-pS15 and γH2AX, both well-established substrates of WIP1 [89]. These data suggest that CCT007093 does not inhibit WIP1 in cells and highlight the urgent need for validation of specificity of small-molecule inhibitors in cellular models including the CRISPR/Cas9-mediated knock-out of the expected target gene.

Compared to previous compounds, SPI-001 and its analogue SL-176 were determined as non-competitive inhibitors of recombinant WIP1 with IC_50_ = 110 and 86.9 nM, respectively [90, 91]. Moreover, SPI-001 was determined to be approximately 50-fold more specific against WIP1 than to another PP2C phosphatase, PPM1A [90]. Both SPI-001 and SL-176 suppressed the cell proliferation in human breast cancer MCF7 cells with overexpressed wild-type *PPM1D* in a dose-dependent manner [91]. In human colorectal carcinoma HCT-116 cells expressing truncated WIP1, treatment with SPI-001 did not affect cell proliferation but combined treatment with SPI-001 and doxorubicin enhanced inhibition of cell growth through the increased phosphorylation of p53 at Ser15 [92]. In conclusion, SPI-001 and SL-176 are promising lead compounds but further analysis is needed to validate their specificity and efficiency in cellular and animal models. Another strategy for development of WIP1 inhibitors was based on modification of short peptides derived from natural WIP1 substrates [8, 93, 94]. Substitution of the pT to pS in the pT-X-pY peptide sequence corresponding to p38 prevented its dephosphorylation by WIP1. Further modification led to development of a cyclic thioether peptide c(MpSIpYVA) with micro-molar inhibitory activity towards WIP1 (Ki = 5 μM). These cyclic peptide inhibitors mimic substrates of WIP1 and block its enzymatic activity in vitro. Further improved cyclic peptide (F-pHse-I-pY-DDC-amide) significantly increased the inhibitory activity and selectivity for WIP1 with Ki = 2.9 μM [93]. The disadvantage of this peptide is poor bioavailability resulting in weak absorption into cells [95]. Therefore, phosphopeptide-based inhibitors have not been tested in cell viability assays to address their anti-proliferative effect. However, the cyclic peptide could be used in future in different drug delivery system, such as nanoparticles.

The most promising compound with high selectivity to WIP1 phosphatase was identified by combination of biochemical and biophysical screens that employed inhibition of WIP1 enzymatic activity and high-affinity binding as readouts, respectively [80]. Both screens identified compounds with overlapping structures containing an amino acid-like core region (referred to as capped amino acids, CAA) flanked by additional groups that influence pharmacokinetic properties [80]. From this series, compound GSK2830371 has been further developed and showed improved cell permeability and pharmacokinetics. According to WIP1 homology model with PPM1A structure and by photo-affinity labeling of WIP1, the binding sites of CAA were located in the Flap domain outside of the active site thus resulting in allosteric inhibition of WIP1. GSK2830371 inhibited WIP1 in vitro with IC_50_ = 13 nM. This compound selectively inhibited WIP1 phosphatase while other 21 phosphatases showed no inhibition of enzyme activity in vitro. Cell proliferation experiments revealed that GSK2830371 efficiently suppressed proliferation of tumor cells carrying *PPM1D* amplification while retaining wild-type *TP53*, including hematological cancer, neuroblastoma, and breast cancer cell lines [57, 80, 89, 96, 97]. Importantly, U2OS-PPM1D-KO cells where *PPM1D* was knocked-out by CRISPR/Cas9 did not respond to GSK2830371 further confirming its specificity to WIP1 at cellular level [89]. Inhibition of WIP1 by GSK2830371 upregulated expression of p53 target genes including *CDKN1A*, *PUMA*, and *BAX* and caused cell cycle arrest but was not sufficient to induce cell death [80, 89, 96, 98]. In addition, GSK2830371 suppressed growth of B cell lymphoma and neuroblastoma in xenograft mouse models demonstrating efficiency of this compound in vivo [80, 96]. Importantly, these studies also demonstrated that GSK2830371 is orally bioavailable. However, relatively low stability of GSK2830371 in blood could limit its clinical use. Further modification of GSK2830371 as a lead compound will hopefully allow development of a small-molecule WIP1 inhibitor with more favorable pharmacokinetic properties.

## Targeting of WIP1 phosphatase in cancer therapy

Restoration of p53 function was shown to cause tumor regression in a mouse model setting ground for development of various compounds capable of inducing the p53 pathway in cancer cells [99]. As described above, inhibition of WIP1 can suppress proliferation of cancer cells by activation of p53 pathway. The highest response is observed in cancer cells with the amplified *PPM1D* (such as MCF7) or truncated WIP1 (such as U2OS), suggesting that these cells might be addicted to the high level of WIP1. In contrast, healthy cells with basal expression of WIP1 are relatively resistant to WIP1 inhibition. Although inhibition of WIP1 strongly suppressed proliferation of cells with high activity of WIP1, it failed to induce massive cell death of cancer cells that would be desirable in cancer therapy [80, 89]. Several studies showed that depletion of WIP1 by RNA interference sensitized cancer cells to DNA damage-inducing chemotherapy [92, 100, 101]. Similarly, GSK2830371 potentiated cytotoxic effect of doxorubicin in breast cancer cells, neuroblastoma, and lymphoma [89, 96, 97]. These results suggest that treatment with WIP1 inhibitor could allow to decrease the efficient dose of doxorubicin and thus reduce its undesired side effects [102, 103]. Similarly, inhibition of WIP1 increased sensitivity of cells to ionizing radiation and to etoposide suggesting that a broader range of potentially beneficial treatment combinations may exist.

Reactivation of the p53 pathway by MDM2 inhibition has been suggested as a promising therapeutic strategy in cancers retaining wild-type *TP53* and several MDM2 antagonists are currently in clinical trials [104–107]. MDM2 antagonist nutlin-3 and its orally bioavailable analogues RG7388 and RG7112 disrupted interaction between p53 and MDM2 leading to stabilization of p53 [108, 109]. MDM2 antagonists efficiently induced apoptosis in p53-proficient neuroblastoma and ovarian clear cell carcinoma and blocked tumor growth in xenograft models [109–112]. Combined treatment with GSK2830371 and nutlin-3 further increased the level of p53 pathway activation and potentiated induction of senescence and apoptosis in MCF7 and HCT116 cells [89, 97, 98, 113]. These data suggest that inhibition of WIP1 that leads to increased phosphorylation of p53 may synergize with compounds that promote stabilization of p53. Besides nutlin-3, other MDM2 antagonists were reported to reactivate p53 and to strongly induce apoptosis of cancer cells, including RITA that binds to p53 at its N-terminus. Whereas the specificity of nutlin-3 has recently been confirmed by CRISPR/Cas9-mediated deletion of p53, cytotoxic effect of RITA was completely independent on the presence of p53, further highlighting the need for validation of the small-molecule inhibitors using modern gene-targeting approaches [114].

## WIP1 activation in p53 negative tumors

As described above, WIP1 is a major negative regulator of p53 pathway. Besides direct or indirect inactivation of p53 pathway, WIP1 was reported to control the expression level of a pro-apoptotic protein Bax through dephosphorylation of a transcriptional factor RUNX2 [115, 116]. This pathway is particularly important in p53-negative cancer cells, where WIP1 activity promotes cisplatin-induced apoptosis. These results led to postulation of an attractive model in which activation of WIP1 can increase sensitivity of p53-negative cells to chemotherapy while protecting the healthy cells (carrying wild-type p53) from possible side effects. However, until now, selective potentiation of WIP1 function remains challenging. One of the possibilities for pharmaceutical intervention could be regulation of WIP1 stability in cells. Turnover of WIP1 in cells is relatively fast (half-life about 90 min) and phosphorylation of WIP1 by HIPK2 potentiates its degradation by proteasome [117]. Indeed, depletion of HIPK2 enhanced the stability of WIP1 and recently has been reported to increase the sensitivity of p53-deficient Saos2 cells to cisplatin [117, 118]. It will be interesting to address the ability of pharmacological inhibitors of HIPK2 to modulate WIP1 levels in cells. Another possibility to increase WIP1 levels in cells might be selective induction of *PPM1D* expression, possibly by RNA-guided activation of endogenous human genes [119]. Clearly, more research is needed to explore suitable approaches for selective WIP1 induction and to experimentally test its benefit for eradication of p53-negative tumors.

## Role of WIP1 in immune response and hematopoiesis

Besides well-established roles of WIP1 in regulation of stress response pathways, there is emerging evidence implicating WIP1 in differentiation of hematopoietic progenitors and in the immune response (recently reviewed in [120, 121]). In particular, *PPM1D* knock-out mice show a p53-dependent block in T cell and B cell maturation in the thymus and bone marrow, respectively [122, 123]. In addition, WIP1 is highly expressed in various kinds of stem cells, and *PPM1D* knock-out mice show increased apoptosis in stem cell compartments [33, 75, 124]. Interestingly, apoptosis in WIP1-deficient intestinal and mesenchymal stem cells was rescued by loss of p53, whereas apoptosis of hematopoietic stem cells (HSC) was p53 independent [33, 75, 124]. Loss of WIP1 led to hyper-proliferation of HSC due to the activation of mTORC1 pathway and led to premature exhaustion of HSC [124]. On the other hand, deletion of p53 rescued the differentiation of WIP1-deficient HSCs into erythroid and myeloid lineages and the repopulation defect in lethally irradiated mice [124]. Finally, mice lacking WIP1 showed increased number of neutrophils and were prone to chronic inflammation such as the DSS-induced colitis [41, 125]. Whereas some of the defects in the immune response observed in WIP1 deficient mice can be explained by abnormal activation of the p53 pathway, others are likely p53 independent. More research is needed to identify molecular mechanisms by which WIP1 regulates the activity of NFκB and mTORC pathways and production of cytokines during the immune response.

## Conclusions and future directions

Data from cell biology and mouse genetics highlight WIP1 as an important negative regulator of p53 pathway and a terminator of the DNA damage response. When overexpressed, WIP1 impairs p53 function and contributes to tumorigenesis, usually in combination with activation of other oncogenes. Conversely, loss of WIP1 significantly delays tumor development in mice and similarly depletion of WIP1 by RNA interference allows reactivation of p53 pathway and inhibits proliferation in p53-proficient tumors. Until recently, specific inhibition of WIP1 represented a major challenge and lack of selective small-molecule inhibitors limited exploitation of WIP1 as pharmacological target in cancer therapy. Situation has changed by development of the compound GSK2830371 that has validated specificity towards WIP1 and efficiently reactivates p53 pathway in various cancer types, including breast cancer, neuroblastoma, and lymphoma. In combination with DNA damage-inducing chemotherapy or with MDM2 antagonists (such as nutlin-3), WIP1 inhibition promotes cancer cell death or senescence, while it has little effect on viability of healthy cells. Importantly, GSK2830371 is orally bioavailable and its ability to suppress cancer cell growth in vivo was demonstrated in xenograft models. In the same time, GSK2830371 is rapidly inactivated in plasma, which may limit its further clinical use. Therefore, further development of GSK2830371 derivatives with more favorable pharmacokinetic properties is highly desirable. Also, solving the 3D structure of WIP1 could stimulate development of even more selective WIP1 inhibitors. Current results suggest that inhibition of WIP1 will be most efficient in cancers with wild-type p53 and amplification or gain-of-function mutations of *PPM1D*, and thus, determination of the status of *TP53* and *PPM1D* in the tumors will be important for predicting the therapeutical outcome of WIP1 inhibitors. Identification of additional factors that control the ability of cells to reactivate p53 pathway is needed to allow prediction of the cancer cell sensitivity to WIP1 inhibitors. MDM2 and MDMX that are commonly overexpressed in tumors seem to be attractive candidates for testing the sensitivity to MDM2 antagonists and WIP1 inhibitors. Although loss of WIP1 is well tolerated in mice, there is emerging evidence that WIP1 plays a role in differentiation of cells of the immune system. In light of these newly arising physiological roles of WIP1, it will be important to address possible side effects of a temporary inhibition of WIP1 during therapeutical intervention.
